# Beyond Organizing Structures: Necessary Conditions to Provide Integrated Care for Youth at-Risk in Practice

**DOI:** 10.1007/s10566-026-09929-0

**Published:** 2026-02-18

**Authors:** Laura C. M. Veerman, Laura A. Nooteboom, Robert R. J. M. Vermeiren, Anne van der Maas, Lieke van Domburgh, Eva A. Mulder

**Affiliations:** 1 LUMC Curium - Department of Child and Adolescent Psychiatry, Leiden University Medical Centre, Post Box 15, 2300 AA Leiden, The Netherlands; 2 Academic Collaborative Youth At-Risk, Post Box 53, Nijmegen, 6500 AB The Netherlands; 3 Lokalis, Joseph Haydnlaan 2A, 3433 AE Utrecht, The Netherlands; 4 Child and Adolescent Psychiatry & Psychosocial Care, Amsterdam University Medical Centre, Meibergdreef 5, Amsterdam, 1105 AZ The Netherlands; 5 iHUB, Mozartlaan 148, 3055 KL Rotterdam, The Netherlands; 6 Child Development and Education, University of Amsterdam, Post Box 15780, Amsterdam, 1001 NG The Netherlands

**Keywords:** Youth at-risk, Integrated care, Qualitative study, High-risk behavior, Mental health

## Abstract

**Background:**

Youth at-risk face multiple and severe problems across various life domains and poses significant risks to themselves and others. The high-risk and high-pressure circumstances and unique needs of youth at-risk make providing integrated care for these youth challenging yet even more crucial. Integrated care refers to coherent, coordinated, and continuous support across services and sectors.

**Objective:**

This qualitative study explores the conditions necessary for providing integrated care for youth at-risk according to professionals, organizational representatives, and policymakers.

**Method:**

Using a grounded-theory approach, we conducted 31 semi-structured interviews with professionals, organizational representatives, and policymakers across four Dutch youth-care regions. Seven observations of multidisciplinary case discussions were used to support triangulation. The study was co-created with stakeholders, including a youth representative who reflected on the findings.

**Results:**

The iterative grounded theory analysis suggests that shared responsibility, shared care coordination, and professional autonomy are key conditions according to stakeholders. Stakeholders perceived that when one of these conditions was lacking, collaboration challenges and stagnation and insufficiency in care were more likely to occur. Stakeholders perceivedthat these conditions mutually influenced one another, such that deficits in one condition can undermine the others.

**Conclusions:**

According to stakeholders, providing integrated care in high-risk contexts involves continuous alignment, collective decision-making, and flexibility among professionals and organizations. Attention to these conditions may help support integrated care practices for youth at-risk.

In recent years, there has been a focus on organizing integrated care for youth with multiple and severe problems across various life domains, including a lack of stability, safety at home and serious mental health issues (e.g., Halsall et al., 2019; Hesjedal et al., 2015). Integrated care is defined as coherent, coordinated, and continuous support, through different levels and sites within the care system (e.g., primary care, specialized care), tailored to youth and their families (Nooteboom et al., 2021). Organizing integrated care consists, for example, of establishing multidisciplinary teams or collaborative network structures, making financial agreements, or merging organizations. However, successfully delivering integrated care for youth requires more than organizing these structures. It requires also conditions such as familiarity, clear roles and responsibilities among stakeholders as well as adequate resources (Cooper et al., 2016; Nooteboom et al., 2021).

Integrated care becomes increasingly important when needs arise across multiple life domains, particularly for youth and their families with multiple and severe risks. A specific group of youth, often aged 12–18 and referred to as youth at-risk, encounter multiple, interacting, and severe problems (Van den Steene et al., 2018). They exhibit high-risk behaviors such as aggression, delinquency, and suicide attempts. This group is heterogeneous and cannot be defined by a single diagnosis or risk factor; these youth often fall between existing services, diagnostic criteria and care structures. The provision of care for youth at-risk occurs under high-risk circumstances, as these youth pose significant risks to themselves and others (Abrines-Jaume et al., 2016). As recurring crises mark the lives of these youth, and the combination of problems and underlying causes differs per case, professionals need to tailor their approach to the specific needs of each youth under intense pressure (Bevington et al., 2015; Malvaso & Delfabbro, 2015; Ungar et al., 2014; Ziviani et al., 2013). Moreover, youth at-risk often mask their problems and have difficulties expressing a need for help due to a lack of epistemic trust. Following Bevington et al. (2015), epistemic trust is defined as a relational process that influences the extent to which information provided by others is experienced as relevant and reliable. This lack of epistemic trust of youth arises from experiences such as a lack of stability at home and a history of treatment failure (Bevington et al., 2015). This lack of trust challenges engaging these youth in care, ultimately creating difficulties to provide integrated care for youth at-risk (De Soet et al., 2023; Munford & Sanders, 2015). Hence, although youth at-risk particularly benefit from integrated care, delivering such care is intricate (Pullmann et al., 2006; Schley et al., 2008; Talbot et al., 2020; Van den Steene et al., 2018).

Providing integrated care for youth at-risk comes with specific challenges. As their needs often vary and require specific expertise, professionals often collaborate with a broad variety of (changing) stakeholders. As a result, there are difficulties regarding familiarity, exchanging information and understanding different perspectives, all important conditions to provide integrated care (Bjønness et al., 2020; Elkington et al., 2020). Studies further outlined that working with these youth is demanding for professionals on an emotional level; they fear for their own physical safety and experience stress and incompetence, resulting in early transfers of youth to other organizations (Okamoto, 2001; Van den Steene et al., 2019). Moreover, professionals and organizations tend to control the high-risk behavior of these youth through punishment, harm reduction, or follow-up assessments instead of treatment or avoid responsibility for treatment out of fear, with no care or referrals as a result (Dagenais et al., 2008; Munford & Sanders, 2015; Veerman et al., 2026).

To our knowledge, we lack insights regarding the provision of integrated care for youth at-risk under high-pressure and high-risk circumstances. Hence, it is vital to understand how integrated care for youth at-risk can be provided in practice. This co-created qualitative study aims to explore which conditions are necessary to provide integrated care for youth at-risk according to professionals, organizational representatives, and policymakers. Insight into these conditions can increase understanding and awareness among professionals, organizations, and municipalities in their journey toward integrated care for youth at-risk.

Based on the reviewed literature, it is expected that providing integrated care for youth at-risk requires more than organizing collaborative structures and is shaped by how professionals and organizations collaborate in practice. It is further expected that challenges related to the unique needs of youth, combined with high-risk and high-pressure circumstances, influence the provision of integrated care by shaping conditions such as familiarity, trust, and role clarity.

## Method

### Study Design

This study is part of the overall action research project “From paper to practice” of the Academic Collaborative Youth At-Risk that started in September 2020. The Academic Collaborative Youth At-Risk is a coalition of experts by experience, youth care services, universities, and municipalities focused on conducting practice-based research. Throughout this project professionals, organizational representatives, policymakers, and a youth representative ensured co-creation by acting as advisors at every stage: setting goals, refining methods, and discussing preliminary findings to guide subsequent research steps. The overarching research project was supported by The Netherlands Organization for Health Research and Development (ZonMw) under Grant Number (70–74700-98–301). The authors declare that there is no conflict of interest and that the funder did not play any role in the conduct and report of this study. The Medical Ethics Review Board of Leiden University Medical Centre judged that the overall research project should not be subject to evaluation based on the Medical Research Involving Human Subject Act (WMO) and complied with the Netherlands Code of Conduct for Research Integrity (non-WMO approval number: N21.108).

In this study, a youth representative additionally reflected on the results (see 4.4). From September until February 2021, an orientation period with open ended interviews (n = 11) and observations of meetings between stakeholders (n = 90) took place and provided the insights that informed this specific study. The input from the orientation phase and co-creation sessions helped shape the research direction. Stakeholder input informed the focus of the study (e.g., topic list), informed the selection of relevant participants, and supported the development of the interview topics. Additionally, various reflexive learning sessions were held during the project. These sessions helped to evaluate the preliminary findings with research participants and relevant stakeholders, and to support development in current practice. Stakeholders validated the preliminary findings and identified key learning points where collaboration was experienced as challenging or where enabling conditions were perceived to be missing in practice. The Consolidated Criteria for Reporting Qualitative Research (COREQ) were used to report the study methods and results (Tong et al., 2007).

## Setting

In the Netherlands, youth care is provided by local community-based support teams, child protection services, specialized care providers and residential care providers. There are 42 youth care regions where municipalities share responsibility for organizing the appropriate care in a timely matter. In this study, we focused on stakeholders involved in organizing integrated care for youth at-risk in four youth care regions. Two initiatives were active in these four regions to provide integrated care for youth. Firstly, a multidisciplinary specialist team consisting of professionals from three residential youth care institutions was operable in these four regions to promote integrated care for youth at-risk. Consisting of eight professionals with diverse expertise (e.g. clinical psychology, coaching, family therapy), the team aims to minimize out-of-home placements and care transfers since 2020. To achieve these ambitions, the team provides consultation and searches for suitable care. To ultimately achieve integrated care for these youth, they must align and collaborate with stakeholders in each youth care region. Secondly, regional tables were organized in two regions in the case that care for youth stagnated: each table had a chair that facilitated and organized meetings between professionals from different organizations.

## Participants

For the semi-structured interviews, we used theoretical sampling, in line with the grounded theory approach (Cutcliffe, 2000). Relevant stakeholders for this study emerged during the orientation phase (interviews therein functioned as ‘gatekeepers’). To obtain regional representation, we aimed to include at least four different stakeholders from each of the four youth care regions. In addition, we interviewed stakeholders from different organizations and with a variety of functions. The overall criterion was that participants had worked as professionals, organizational representatives, or policymakers for youth at-risk. We defined professionals as ‘respondents who regularly worked with youth at-risk in care’ (e.g., therapist), organizational representatives as ‘respondents who had primarily tasks to coordinate other professionals who provide care for this group’ (e.g., chair, manager), and policymakers as ‘respondents who worked at a municipality for policy development regarding youth at-risk’. Based on the orientation phase, eligible participants were invited to participate in a semi-structured interview via email by the interviewer (LV). In the invitation, participants were informed of the study's objectives and interview procedure and subsequently had to provide a written informed consent before participating in the interview. No incentives were provided. Five respondents did not respond, and one respondent declined without reason, leading to approaching other respondents within the same organization and region. Ultimately 37 eligible participants in total were approached, of whom 31 actually participated. As theoretical saturation seemed to be achieved within the information of these 31 participants (see Analysis), there was no further need to recruit more participants (Saunders et al., 2018).

## Data Collection

Semi-structured interviews were conducted between May and September 2021 by one (LV, n = 21) or two interviewers (LV, OS, n = 10). Based on the respondent's preference, interviews were scheduled either online via Microsoft Teams (n = 24) or at the participant’s workplace (n = 7). The interviews were guided by a topic list with open-ended questions to facilitate a deep understanding of the viewpoints and experiences of stakeholders. The topic list was formulated in advance, based on the orientation phase and previous research (Bronstein, 2003; Nooteboom et al., 2020, 2021). The topic list was then supplemented with input from reflection meetings with professionals, policymakers, youth representatives, and multiple researchers. After that, it was pilot-tested on one respondent who met the inclusion criteria. The topics focused, for example, on the trend of multidisciplinary teams, trust, professional autonomy and preconditions for collaboration (see Appendix A). All interviews were conducted in Dutch, audio-recorded, and transcribed verbatim to prevent interpretation bias. Two researchers (LV, KD) translated the quotes from Dutch to English. The length of the interviews ranged from fifty minutes to two hours (m = 71.1 min). None of the participants expressed an interest in commenting on the transcripts. In January 2022, a learning session was organized with the interview participants and their colleagues (n = 21), as well as a youth and parent representative. The goal of this session was to reflect on and learn from the preliminary results of the interviews. This session also served as a member check. In addition, open observations of clinical case discussions of the multidisciplinary specialist team (n = 68) were conducted by one researcher (LV) in the overall research project from January 2021 to June 2022. These observations provided an insight into what professionals in this team encountered in practice when providing integrated care for youth at-risk. For this study, we randomly selected seven of these observation reports of clinical case discussions.

## Reflectivity

As part of the overall research project, the first author was familiar with the way of working and the stakeholders involved in the research setting (see 2.2). As a result of becoming acquainted with some of the respondents and the expectation that they would be more open due to shared experiences, some interviews were deliberately conducted solely by the first author. The researcher wrote reflections before and after the interview to increase self-awareness and prevent bias. Moreover, weekly reflective meetings were held with members of the research team to discuss these reflections and prevent observer and interview bias (LV, LN, EM). These reflection notes and meetings also supported the theory-building process in each step, as described below.

## Analysis

All interview transcripts were imported into the computer program ATLAS.ti (version 9) for coding. We used a grounded theory approach for analysis (Strauss & Corbin, 1990), as it allowed for a deeper exploration of local and personal processes and experiences (Cutcliffe, 2000; Foley & Timonen, 2014). The analysis consisted of three iterative steps. Firstly, the interviews were open coded by one researcher (LV) by assigning keywords to parts of the text inductively in combination with the reflection notes. These open codes were constantly compared to new data. Open coding was conducted manually, after which a preliminary codebook was created to explore similarities. This process was iterative, with codes being continuously refined and grouped as analysis progressed. Secondly, axial coding was conducted to examine relationships between codes that had been developed in the open coding phase by comparing parts of the text with the same code for differences and similarities. This step was supervised by an experienced qualitative researcher (LN). Codes were thereby split and combined, and new codes were identified, allowing to describe categories within the theory in more detail. Decisions regarding the grouping, splitting, or merging of codes were discussed during regular team meetings, and evolving coding frameworks were shared and discussed within the research team. After axial coding of 20 interviews, no new codes were identified, indicating inductive thematic saturation was reached (Saunders et al., 2018). Saturation was achieved across stakeholder perspectives, as no new codes emerged in subsequent interviews regardless of participants’ role, and later interviews confirmed existing codes. Based on the first two steps, an inductive coding framework was built to code all interviews. Third and finally, based on the coding of all interviews and examining the relationship between codes through constant comparison, the research team abstracted three core categories that formed the main theory. Throughout this process, analytic interpretations were discussed in weekly research team meetings, during which consensus was reached through discussion. After analyzing the interviews, seven randomly selected observation reports of clinical case discussions (out of 68 reports in total) were analyzed to enrich the theory. The observation reports were inductively and deductively analyzed based on the theory that emerged through the interview analysis. The purpose of this analysis was to examine whether and how this theory emerged in the observations and to further illustrate and contextualize the interview findings. As no additional categories emerged from the observation reports, they were used to confirm and enrich the core categories identified in the interviews. The seven observation reports reflected variation across cases, stakeholder involvement, and different moments in time. As the core categories were confirmed in these reports, variation of cases were present and no new themes were identified, no additional observations were analyzed. This final step of theory building, allowed us to refine the core categories and the relationships between each category.

## Results

### Demographics

A total of 31 respondents were interviewed. Table 1 provides a summary of the participants' demographics. Most of the participants were female (n = 28), which represents the gender distribution of stakeholders in youth care in the Netherlands. The largest group of participants were members of a multidisciplinary team, as this was the focus of the overall research project. Overall, the participant groups consisted of professionals who regularly worked on a clinical level with youth at-risk in care (e.g., therapist, behavioral scientist), organizational representatives whose primary tasks were to coordinate professionals that provide care for this group (e.g., chair regional table, manager, coordinator), and policymakers who worked at a municipality for policy development regarding youth at-risk.

**Table 1:** Demographics of interview participants

Variable	
Gender	
Male [n (%)]	3 (9.7%)
Female [n (%)]	28 (90.3%)
Age	
Mean age in years [SD]	43.5 (10.38)
Age range in years	27–62
Years of work experience in current organization	
Mean in years [SD]	3.25 (4.11)
Range in years	0.17–21
Organization*	
Municipality [n (%)]	4 (12.5%)
Community-based support teams [n (%)]	6 (18.75%)
Child protection services [n (%)]	5 (15.6%)
Regional table [n (%)]	2 (6.25%)
Specialistic youth care providers [n (%)]	3 (9.4%)
Multidisciplinary specialist team [n (%)]	9 (28.1%)
Residential youth care organizations [n (%)]	3 (9.4%)
Function	
Community-based support team worker [n (%)]	2 (6.5%)
Behavioral scientist community-based support team [n (%)]	4 (12.9%)
Therapist specialist care [n (%)]	2 (6.5%)
Behavioral scientist child protection service [n (%)]	5 (16.1%)
Consultant multidisciplinary specialist team [n (%)]	8 (25.8%)
Coordinator treatment residential care [n (%)]	3 (9.7%)
Chair regional table [n (%)]	2 (6.5%)
Manager [n (%)]	1 (3.2%)
Policymaker [n (%)]	4 (12.9%)
Division per region	
Region 1	4 (12.9%)
Region 2	4 (12.9%)
Region 3	4 (12.9%)
Region 4	6 (19.4%)
Supra-regional	13 (41.9%)

^*^One respondent worked in two organizations at the time of the interview with similar functions, both relevant to this study. Therefore, the number of respondents adds up to 32 in the description of organizations

## Findings

**Fig. 1:**
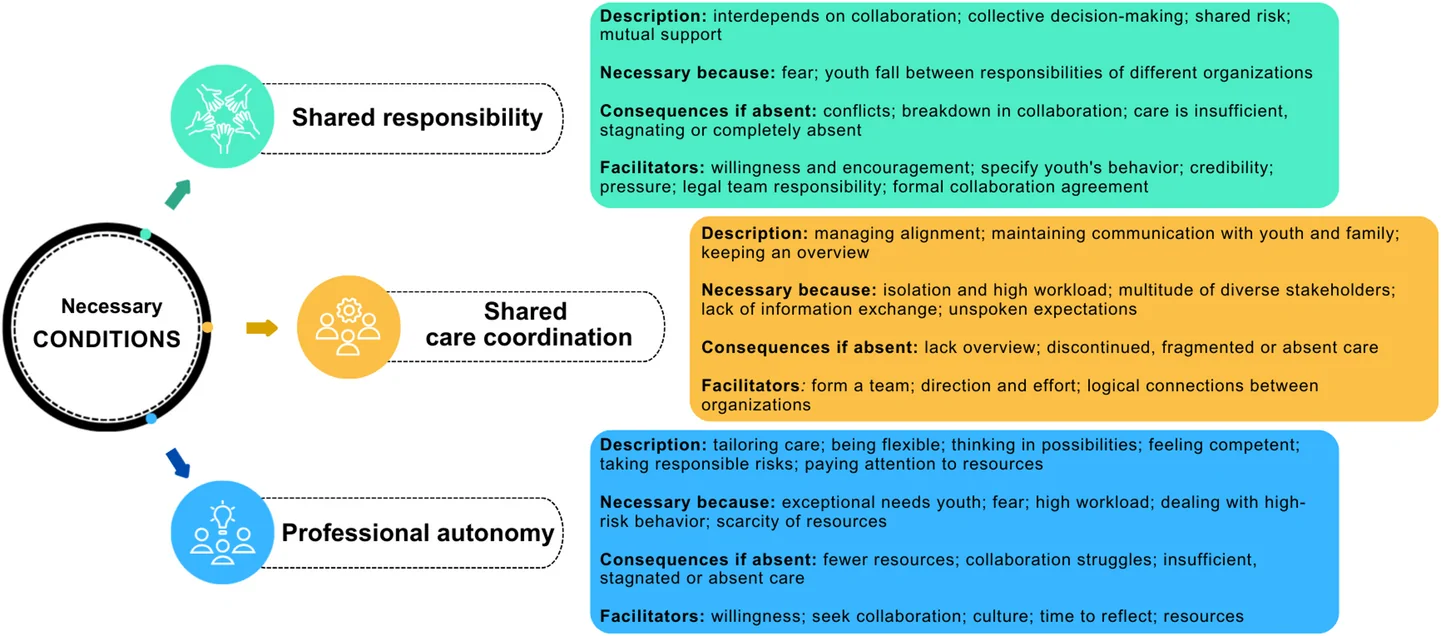
Overview of results

Based on our analysis, we constructed a theory in which three conditions are deemed necessary according to professionals, organizational representatives, and policymakers when providing integrated care to youth at-risk: shared responsibility, shared care coordination, and professional autonomy. For each condition, we describe what it entails, explain why this condition is necessary in practice, illustrate the consequences of its absence in practice, and discuss facilitators for its realization. Finally, the three conditions seem to interrelate with each other, as outlined in the final paragraph. The described results are based on the analysis of the interviews, with additions from the observation reports explicitly mentioned. Figure 1 presents an overview of the results.

### Shared Responsibility

Because that responsibility is huge. Imagine that a child commits suicide, while you are the supervisor there, no way. I don't provide the care, just go to the next counter. (…) And if you're responsible, you're screwed. That's pretty clear. — Respondent multidisciplinary specialist team 2.

The first condition is shared responsibility (n = 26), which means that stakeholders share responsibility between several organizations to provide care for youth at-risk. Responsibility does not lie with one professional or organization but with all stakeholders involved. Shared responsibility interdepends on collaboration, as multiple organizations and professionals need to be involved in caring for these youth due to their diverse needs. Accordingly, it is crucial to collectively make decisions, leading to a sense of shared risk and mutual support. This is also underscored in the observations (n = 3).

However, more than half (n = 19) of the respondents mentioned that stakeholders often make unilateral decisions to either refuse responsibility or force responsibility onto others. This is also supported by the observations (n = 3). Our analysis provided two explanations for not sharing responsibility in care for these youth in practice. Firstly, the reluctance to take and share responsibility and the tendency to reject or pass on cases to others seem to stem from fear (n = 15). Respondents mentioned they prefer to avoid risks, as there is pressure from the media, municipalities, and caregivers. Additionally, the consequences could be significant if things go wrong, such as a fatal suicide or severe aggression. Professionals can also be held personally accountable under disciplinary or criminal law; therefore, they are more likely to avoid taking responsibility or to force responsibility until it is taken. Secondly, conflicts exist regarding who is responsible, as this target group falls between the responsibilities of different organizations and legal frameworks, such as juvenile law and various procurement contracts between municipalities and care providers (n = 16). This is also underscored in observation reports (n = 5). Separate organizations refused to take responsibility for the diverse needs of these youth in combination with their high-risk behavior. They deemed themselves ineligible for these specific services, as it exceeds the capability of their organization. Conflicts between stakeholders easily occur as the involved stakeholders and organizations all approach the needs of youth at-risk from different perspectives and interests and as they are under much pressure.

What we run into is that behavioral scientists from [names of organizations] say; ‘that’s not for us’, and to the offering party you say, ‘figure it out’. Taking responsibility together, that’s not really there yet. More and more often they simply say ‘we’re not going to do that’, with the greatest ease. — Professional during observation 57.

Consequently, respondents experienced unilateral decisions to avoid or force responsibility onto another stakeholder as a breakdown in collaboration between stakeholders: they felt they were not taken seriously. This was associated with feelings of distrust towards the stakeholders that they depend on in providing integrated care in practice (n = 5). This is also underscored in one observation. For youth, these breakdowns in the collaboration resulted in rejections by care organizations, multiple transfers between providers, fragmentation of care, or even complete stagnation due to a lack of available services according to respondents. Some respondents described that they sometimes feel they are “endlessly” searching for organizations to take responsibility. In some cases, respondents responded to this lack of responsibility by taking on tasks they were not equipped to do. Nevertheless, one respondent mentioned that she preferred not to share responsibility with others, and continue to make unilateral decisions. She described that care should be provided by her organization, as this is the best way to help these youth.

Six facilitators were described as supporting shared responsibility when providing integrated care for youth at-risk. Firstly, stakeholders need to be willing and daring to share responsibility, which can be encouraged by personal relationships among stakeholders (n = 13). Municipalities can facilitate meetings between different stakeholders to support professionals and organizations in making collective decisions and encourage connections between stakeholders to take shared responsibility (n = 10). In this regard, three observations revealed it is important to show willingness, and be clear about which responsibilities each professional and organization can take to prevent friction and confusion. Secondly, it is important to specify the behavior of youth at-risk to prevent the wrong organization, or no organization, from taking shared responsibility, especially during a crisis or when a young person runs away (n = 4 observations). This requires a thorough understanding of each youth's specific needs to determine who should take responsibility. It helps to know that these behaviors are common among youth at-risk and necessitate time and a long-term perspective on care.

The term running away gives a burden, and professionals start to get restless. I had a conversation with someone the other day, that someone was walking back home. I don't think that's running away. However, that does appear in the files as running away. – Professionals about youth during observation 39.

Thirdly, find each other credible facilitates shared responsibility (n = 14), meaning they can trust each other's judgment and are able to be transparent with each other about the responsibility they can take. Credibility increases according to respondents when stakeholders know each other and have years of experience working together and working with youth at-risk. Fourthly, six respondents described stakeholders need some pressure to share responsibility. Ways in which pressure can be effectively exerted include, for example, “we will not leave the table until a solution is found” or “removing” the ability of teams to say ‘no’ by shifting the decision-making power to another team in an organization. Fifthly, three respondents noted that instead of individuals bearing sole legal responsibility under disciplinary law, it would be beneficial for organizations or the team surrounding a specific child, to share this legal responsibility. This shared approach could help prevent professionals from avoiding responsibility due to fear of personal legal consequences. Sixth and lastly, a formal collaboration agreement between organizations makes it more difficult for the organizations to refuse responsibility (n = 5).

### Shared Care Coordination

I find it quite complicated in the collaboration between, for example, the community-based support team, child protection service, and care provider. Because then you are basically already with three parties, all three of which have a certain degree of care coordination on what is needed here, what direction do we choose? What is the perspective? Who does what in this? — Respondent multidisciplinary specialist team 7.

The second condition is shared care coordination. This entails managing alignment among all stakeholders involved, including task and role division, maintaining continuous communication with the youth and family, and keeping an overview of the provided and required care (n = 19). Shared care coordination for youth at-risk is a joint action and lies with multiple stakeholders in a care process. Shared care coordination requires regular meetings for monitoring and evaluation, during which information is exchanged, decisions are made, and tasks, roles, and expectations are explicitly stated, to ensure that care remains aligned and relevant stakeholders stay involved (n = 29). This condition is also explicitly emphasized in three observations.

Although, in most cases, it is clearly defined on paper who has what task and role, care coordination for youth at-risk is often lacking in practice. Our analysis provided four explanations for the lack and necessity of shared care coordination when providing integrated care for youth at-risk. Firstly, seven respondents noted that professionals who individually take on care coordination for these youth often feel isolated and experience a high workload, emphasizing shared care coordination. Secondly, there is a multitude of (changing) professionals involved due to the diversity of problems faced by youth at-risk, leading to a lack of overview of who is involved and who has which role in the care process. Respondents described this could lead to uncertainty and conflicts regarding role and task division (n = 18), but also in overlooking important stakeholders (n = 4 observations). Thirdly, two observations underscored the difficulty of exchanging information and alignment between multiple stakeholders, given their perception of the General Data Protection Regulation. Fourth and lastly, respondents (n = 12) mentioned expectations of each other often remain not communicated or are wrongly assumed (n = 3 observations). Respondents described this could result in the youth and their family receiving different messages and fragmented or no care, as stakeholders assume and might even prefer someone else taking on a task.

People very quickly tend to think ‘[name of organization] is involved so we don't have to do anything anymore’. – Professionals during observation 17.

Accordingly, respondents perceived a lack of shared care coordination as related to challenges in relational continuity of care, distrust from youth and their families, a lack of overview, and fragmented or absent care (n = 10 interviews, n = 2 observations). Based on our analysis, three facilitators were described to support shared care coordination in practice, Firstly, stakeholders need to explicitly state at the outset that shared care coordination is a joint action, and that all stakeholders must form a team for each specific case (n = 5). Secondly, it requires direction and effort from all stakeholders involved to commit to shared care coordination; each must initiate meetings when necessary. It helps if one stakeholder takes the lead in planning and facilitating meetings (n = 6). Finally, municipalities play a crucial role in underpinning shared care coordination: they are responsible for organizing (an overview of) the care landscape and facilitating logical connections between stakeholders (n = 8).

### Professional Autonomy

And yes, there is a young boy who is in our group, but who actually can't stay in that group because he gets so incredibly over-stimulated and it's really harmful to him. We happen to have an empty building. (…) And we also said: ‘Yes, we can just make use of that building, because if he gets one-on-one care and he does quite well at school. Yes, then we can continue to provide care for him for a long time. And then you can also really tailor that treatment in a different way. Only we really don't have any staff. (…) And that makes it complicated because then you can have good solutions with all the good intentions. And then you do have a mandate to organize that. And we can also often use [funding opportunity]. So, a lot is possible. Only you run into the limits of staff and buildings. – Respondent residential youth care 2.

The third condition is professional autonomy, which means the autonomy stakeholders experience to tailor support to the needs of youth at-risk, while taking responsible risks if needed (n = 21). According to respondents, professional autonomy includes stakeholders being flexible and creative, thinking in terms of possibilities instead of standard procedures, searching for collaboration to deliver tailored care and feeling competent in taking responsible risks by adapting existing services. Moreover, it also comprehends paying attention to sustainable resources, such as staff, buildings, and funding. This condition is underscored in two observations.

Nevertheless, there is often a lack of professional autonomy in practice. Our analysis revealed five explanations for the lack of and urge for professional autonomy. Firstly, as youth at-risk often encounter crisis situations, they form the exception in youth care, requiring a lot of time to find available and tailored care (n = 15). For example, in one observation, organizing funding required so much time that too little attention was paid to the youth's immediate needs. Another observation showed that the time needed to organize the exceptional care led to further escalation of a youth's problems. Secondly, respondents described stakeholders remain focused on standard procedures and organizations out of fear of making mistakes with fatal consequences, and they, therefore, find it difficult to be flexible towards the problems of youth at-risk (n = 3). As a result, respondents described the care is not tailored to the needs of youth at-risk. In one observation, professionals mentioned they struggled to collaborate with others who are focused on standard procedures, as youth at-risk often do not fit the standard.

Because that's where it gets stuck every time, that people don't want to get involved in such a complex case because of the risks of suicides. (…) Well, you know, you can have all kinds of risks of things going really wrong, where the inspection gets involved. (…) People are careful (…). They're not going to push the boundaries. – Respondent multidisciplinary specialist team 4.

Thirdly, a high overall workload leads to stakeholders feeling that they have limited capacity to tailor care to the needs of these specific youth (n = 4). Fourthly, professionals struggle to deal with the high-risk behavior (aggression, walking away) of youth at-risk. Consequently, respondents described professionals drop out of work, which in turn results in fewer staff to provide care for youth (n = 5). Finally, limited resources are available to organize the often expensive and diverse combinations of care for youth at-risk (n = 17; n = 2 observations). As a result, respondents described organizations as less flexible in adapting to these youths’ problems, and reported that municipalities and organizations were unable to organize care for this group.

However, stakeholders find it difficult to admit they cannot provide the expected quality of care when lacking resources, as they feel compelled to stay operational. Additionally, expectations from municipalities and other parties to provide tailored care for youth at-risk are high. These circumstances were associated with organizations providing care ineffectively and with a perceived loss of credibility and trust (n = 5). These cases can initiate a negative spiral in which teams continue to provide care despite stating their inability to do so, ultimately leading to teams dropping out, with even fewer resources available and youth stagnating or not receiving care (n = 6; n = 4 observations).

What they are struggling with is that she [young person] has been occupying the only place at [name organization] for months and therefore they have to refuse other young people who also need care. It's also a sort of ethical dilemma. – Professionals during observation 39.

Hence, respondents noted that limited professional autonomy was associated with difficulties in providing tailored care, fewer available resources, and challenges in collaboration. Five facilitators for professional autonomy are outlined based on the results. Firstly, municipalities, organizations, and professionals need to be willing to create tailored care (n = 9).

I think that it is also why they [youth at-risk] fall between the cracks everywhere. Because all these products actually just don't fit them. So, you have to be willing to make them fit. – Respondent multidisciplinary specialist team 6.

Secondly, when organizations seek collaboration with each other and look at their shared resources, opportunities arise to jointly shape integrated care (n = 5). A collaboration agreement between organizations facilitates combining shared resources. For example, a collaboration agreement enabled organizations to approach resources as collective rather than organization-bound, making it easier to explore tailored solutions for youth at-risk. Thirdly, a culture where flexibility, out-of-the-box possibilities, and customized solutions are jointly explored, also in high-risk situations, can facilitate professional autonomy (n = 19). This culture requires trust and creativity instead of control. It also comprehends a demand-oriented rather than supply-oriented approach from all stakeholders involved. This is also emphasized in one observation. Municipalities and organizations can support this culture by encouraging professionals to think in terms of possibilities and by letting go of standard procedures. For example, one respondent described how close and direct lines of communication with the municipality enabled swift decision-making based on professional judgment. In this situation, resources could be deployed quickly and flexibly, allowing professionals to prioritize the needs of the youth without being constrained by procedures or administrative processes. Fourthly, tranquility and time to reflect, even when they are under high pressure, can help stakeholders explore out-of-the-box solutions (n = 8; n = 1 observation). Finally, sufficient resources facilitate professional autonomy, such as finances (task-oriented funding), time, and staff (n = 8; n = 1 observation).

### Interrelatedness Between the Conditions

Based on our results, the beforementioned conditions interrelate with each other, both positive and negative. The positive influence between conditions means that when one condition is present, it can facilitate the presence of another condition. For example, shared responsibility encourages mutual support and stakeholders to be more understanding towards each other. This openness was associated with a broader perspective and focus on exploring shared resources and possibilities. This means that shared responsibility between stakeholders can also facilitate more professional autonomy (n = 7; n = 3 observations). As one respondent describes:

And look, the moment you get much more into that collaboration and start working together, you have shared responsibility and you often also become a little easier on each other and a little more gentle. So, then you are also willing to just look with a broader view and just look at, hey, what are the solutions and the possibilities instead of impossibilities. – Respondent specialistic youth care provider 1.

The negative influence between conditions means that when one of the three conditions is missing, this can lead to the absence of another condition. For instance, stakeholders can lack resources and time to tailor care to the needs of youth due to high-pressure circumstances. Consequently, stakeholders refuse to provide care and force responsibility onto others according to respondents, illustrating how a lack of professional autonomy also creates a lack of shared responsibility (n = 4; n = 1 observation).

## Discussion

This qualitative study explored which conditions are necessary to provide integrated care for youth at-risk according to professionals, organizational representatives, and policymakers. A small group of youth characterized by high-risk behavior which often form the exception in care. Based on a grounded theory analysis of 31 semi-structured interviews supplemented with seven observation reports of clinical case discussions, we formed a theory in which three interrelated conditions were identified as necessary and thus require explicit attention in practice: shared responsibility, shared care coordination, and professional autonomy (see Fig. 1).

These conditions resonate with earlier studies on families with multiple and enduring problems (e.g., Nooteboom et al., 2020) and research focusing on youth at-risk and their families, which have highlighted the importance of sharing responsibility (e.g., Dagenais et al., 2008; De Soet et al., 2023), coordinating care (e.g., Fraser et al., 2016; Ungar et al., 2014), and professional autonomy (e.g., Malvaso & Delfabbro, 2015; Willumsen & Hallberg, 2003). Our study is complementary as it illustrates the in-depth insights regarding the experiences of a diverse group of respondents (professionals, organizational representatives, policymakers) when providing integrated care for a specific group of youth under intense pressure and high-risk circumstances. By outlining why these conditions are often difficult to achieve in current practice according to stakeholders and describing the facilitators that help their realization, this study contributes to existing knowledge and provides insights for clinical practice to improve the working approach for youth at-risk.

In this section, we discuss three key findings and its implications. Firstly, providing integrated care for youth at-risk requires stakeholders to focus on these conditions by encouraging flexibility and embracing complexity as it enables them to address the evolving and unpredictable needs of youth at-risk. Secondly, stakeholders' fear of making mistakes and the perceived risk of fatal consequences can impair necessary conditions, resulting in stagnation or a lack of care. This is why integrated care should be a stable team journey with continuous alignment between stakeholders. Lastly, building trust among stakeholders is essential to prevent overburdening and inadequate care provision in high-risk and high-pressure circumstances, thereby ensuring shared responsibility and professional autonomy. Additionally to these key findings, reflections of a youth representative (AM) based on our study’s results are outlined to incorporate the perspective of youth on these results.

## From “Makeability” to “Complexity”

As our results demonstrated, shared responsibility, shared care coordination, and professional autonomy are necessary to provide integrated care according to professionals, organizational representatives, and policymakers. Nevertheless, high expectations of organizations and high-risk situations put pressure on organizations to quickly provide integrated care, which were associated with inadequate and unsuitable care, as well as struggles in collaboration. This sheds light on a broader issue, namely makeability thinking. Makeability thinking, also known as “simplification”, is known for the mentality of minimizing risks, increasing efficiency, and processes are rational and followed by a clear protocol (Van Putten, 2023). From this perspective, makeability thinking may be considered as limiting efforts to provide integrated care, whereby a focus is on, for example, organizing a team or collaborative structure, and little attention is paid to capability of stakeholders. Makeability thinking does not fit the needs of youth at-risk: their needs form the exception in youth care, which makes following a clear protocol difficult. Stakeholders must look beyond organizational structures to provide integrated care for youth at-risk. Instead, complexity thinking might provide an alternative to support the provision of integrated care for youth at-risk. Complexity thinking, also known as “complexification,” emphasizes coherence and continuous alignment between stakeholders, with processes emerging and adapting to unpredictable and uncertain circumstances (Joosse, 2022). As the needs and behavior of youth often change and form exceptions, complexification can support professionals, organizations, and policymakers. Complexity thinking inherently requires shared responsibility, shared care coordination, and professional autonomy: equal attention to stakeholders' obligations, as well as, their possibilities, with respect for and alignment of capability limits to prevent negative outcomes. Ultimately, beyond organizing structures, complexity thinking practically implicates alignment through regular meetings, monitoring and evaluations.

## A Stable Team Journey Toward Providing Integrated Care

The results of our study emphasized stakeholders’ fear of making (fatal) mistakes and being isolated or held accountable. This fear can result in the absence of shared responsibility, shared care coordination, or professional autonomy. This in turn leads to stagnation, inappropriate support, or a lack of care. Therefore, we suggest that stakeholders should have a team focus across organizations rather than focusing solely on their own organization. This focus can facilitate the presence of these conditions, thereby providing integrated care. By building a stable team, stakeholders with different expertise and from various contexts can experience less fear, feel more committed and supported, share responsibility, and take joint action (Dagenais et al., 2008; Van den Steene et al., 2018). As each case is different and team compositions change with varying circumstances, this requires the stakeholders involved to monitor progress through regular meetings, ensuring that they remain aligned and connected with one another.

## Building Trust

As outlined in our study, a lack of shared responsibility and professional autonomy in high-risk and high-pressure situations can lead stakeholders to provide care or perform tasks they are unable to deliver adequately. This can occur because organizations, despite stating their limitations, feel pressured to provide care due to high expectations. When care is ineffectively provided, this can result in distrust among stakeholders in each other’s judgment, as stakeholders feel they are not taken seriously. Our results have illustrated that a lack of trust can create a negative spiral in which teams drop out due to the overburdening of the care they must provide, ultimately leading to fewer resources and insufficient care for youth at-risk. These results shed light on the relational process of epistemic trust: the belief in the relevance and reliability of information provided by others. Previous studies have already emphasized the cruciality of epistemic trust between youth and professionals for treatment success (Bevington et al., 2015; De Soet et al., 2023). Our findings suggest that epistemic trust may also play a role among stakeholders (Bevington et al., 2015). When stakeholders lack trust in each other’s judgment, collaboration becomes difficult, and care for youth can be negatively affected. Therefore, building trust among stakeholders is crucial to facilitate shared responsibility and professional autonomy, thereby providing integrated care for youth at-risk. Future research is warranted to explore how trust can be built between stakeholders. For current practice, the triple C method might be helpful which stands for client (i.e. youth at-risk), coach (i.e. professionals), and competence. This methodology has, as its premise, that each person, whether it is a client or a professional, has the same needs. It focuses not only on how to build trust with the client but also amongst stakeholders in order to provide care to challenging groups (Tournier et al., 2020).

## Reflections from a Youth Representative

**Fig. 2:**
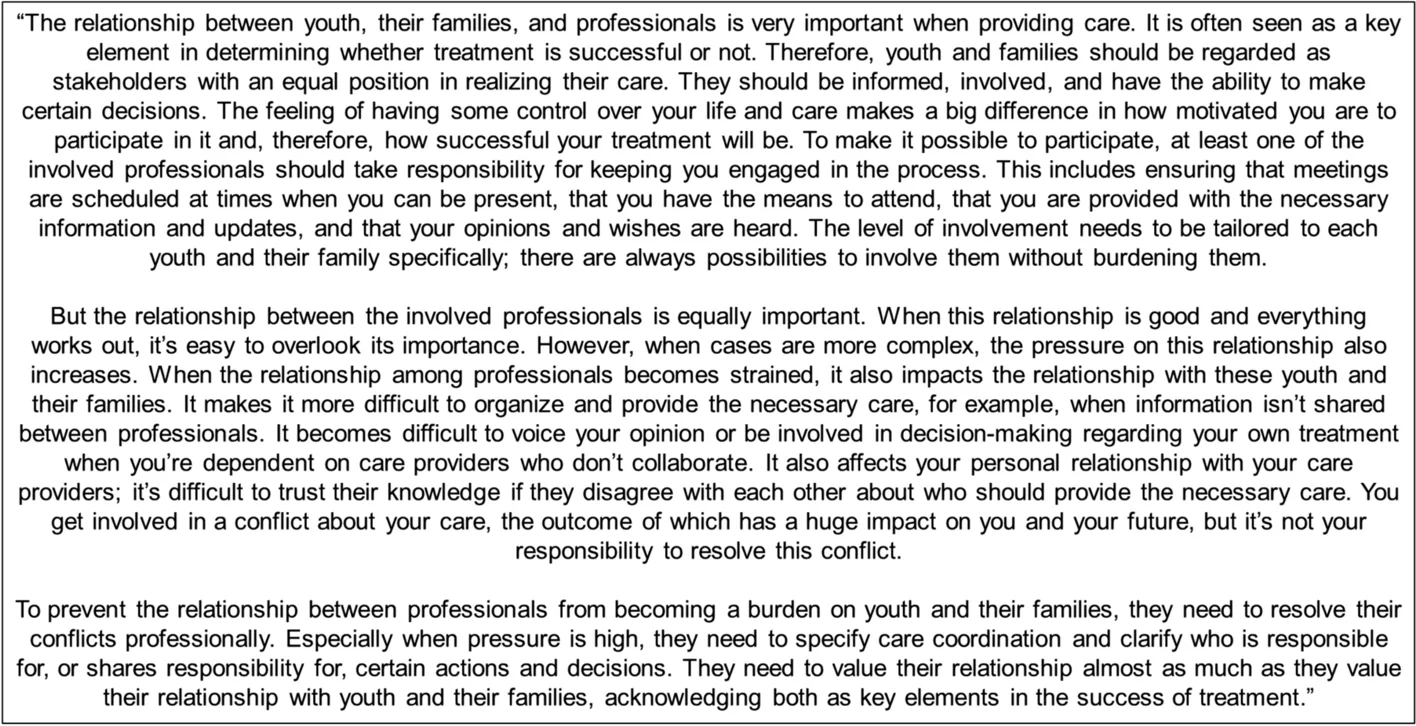
Practical implications from a youth representative

In Fig. 2, a youth representative reflected on the results of this study, as our results were based on the experiences of professionals, organizational representatives and policymakers.

## Strengths and Limitations

The qualitative, and explorative nature of this study allowed us to enrich insights to build a theory on what is needed to provide integrated care for youth at-risk. This study contributes to existing knowledge by providing a deeper understanding of these conditions in this specific context, their facilitators, and their interrelatedness when providing integrated care for youth at-risk. The relevance of this study for current practice was strengthened by the co-creation process with professionals, policymakers, and youth representatives in the overall research project. Moreover, the conditions found in this study were consistent in the four regions and across a diverse range of respondents from various organizations and functions, thereby increasing credibility and applicability (Watkins, 2012). We further described our research following the COREQ checklist, improving transparency of our results (Tong et al., 2007).

Nevertheless, our study also comes with several limitations. Firstly, one limitation concerns potential threats to the trustworthiness of the findings. Although quotes were translated with careful review by multiple researchers and observation reports were randomly selected from a larger dataset, some loss of nuance and limited contextual variation cannot be ruled out. Secondly, the findings are situated within the Dutch youth care system, which is characterized by regionally organized governance and contract-based service delivery. This shapes how collaboration between professionals, organizations, and policymakers is organized and experienced in practice. Accordingly, their transferability to other settings requires careful consideration of local governance structures, service arrangements, and population characteristics. The findings of this study may be understood as potentially transferable in the sense that they provide contextually grounded insights into collaborative practices that may be relevant to other settings facing similar challenges, while requiring adaptation to local conditions (Stalmeijer et al., 2024). Future research could enhance transferability by examining the conditions for provide integrated care for youth at-risk across different youth care contexts, thereby distinguishing context-specific conditions from those that may be transferable across settings. Thirdly, although the identified conditions were consistent across regions and respondents, the findings should be interpreted in light of the diversity of service contexts and the heterogeneous needs of youth at-risk. Differences in socioeconomic circumstances, cultural and linguistic backgrounds, geographic settings (e.g., rural versus urban contexts), and disabilities may affect the feasibility of realizing shared responsibility, shared care coordination, and professional autonomy in practice. Fourthly, performing a grounded theory approach analysis could potentially be biased, since interpretation can be influenced by personal experience and knowledge of the researcher familiarity with working in this particular setting. To prevent such bias, reflexive notes, meetings, and research team discussions were conducted (Choy, 2014).

Another limitation is that we did not include the perspectives of youth and their families in this study as we focused on multiple and diverse perspectives of professionals, organizations, and policymakers. Nevertheless, the experiences of youth and their families are crucial to determine whether integrated care is provided. Therefore, future research is needed to examine the identified conditions on a clinical level by following multiple youth at-risk cases. This would enrich existing insights into what this requires of youth and their families, and how stakeholders should approach both them and each other when providing integrated care.

## Conclusion

This co-created qualitative study has identified three interrelated conditions necessary for providing integrated care for youth at-risk: shared responsibility, shared care coordination, and professional autonomy. The absence of these conditions can lead to stagnated, fragmented, or absent care for youth and their families, as well as difficulties in collaboration among the involved stakeholders. Therefore, these conditions require explicit attention in practice through flexibility, embracing complexity, maintaining a team focus, and building trust among stakeholders to provide integrated care for youth at-risk in high-risk, high-pressure circumstances.

## Data Availability

Two authors had access to the data. One author (LV) takes responsibility for the integrity of the data and the accuracy of the data analysis.

## Appendix

See Tables

**Table 2:** Topic list professionals and organizational representatives

Topics	Questions
Background information	- Age, years working in this function and organization
Trend of multidisciplinary teams	- Improving integrated care for youth at-risk and families is often sought by organizing a new structure, such as in the form of a new multidisciplinary team. Do you recognize this development? - What is your perspective on this development? - Suppose you could create a name for a new team that reflects what you believe is needed when professionals face challenges with a youth at-risk case. What would it be called, and what would this team do?
Preconditions to collaborate	- In your opinion, what is needed in collaboration between stakeholders ensure that youth and families with the most complex issues receive appropriate, cohesive support?
Taks and role division	- How are the tasks and responsibilities coordinated between stakeholders in practice? - Is it clear who has which task in collaboration between stakeholders?
Responsibility	- How does the responsibility of other stakeholders relate to the responsibility of your own? - To what extent do you experience shared responsibility in collaboration with stakeholders in practice? Do you find it important to experience this?
Trust expertise	- To what extent do you have trust in the expertise of stakeholders? - To what extent do you perceive trust in your own expertise from these stakeholders?
Professional autonomy	- To what extent do you have the capacity to act on the advice of other stakeholders? For example, when it comes to funding custom solutions instead of purchased care
Reflection interview	- Finally, would you like to add anything? - How do you feel about the interview?

2,

**Table 3:** Topic list policymakers

Topics	Questions
Background information	- Age, years working in this function and organization
Role municipality in relation to realizing integrated care for youth at-risk	- How do you see the role of the municipality in realizing integrated care for youth at-risk? - What facilitates or hinders this?
Trend of multidisciplinary teams	- Improving integrated care for youth and families is often sought by organizing a new structure, such as in the form of a new multidisciplinary team. Do you recognize this development? - What role do municipalities play in this development? - What is your perspective on this development? - Suppose you could create a name for a new team that reflects what you believe is needed when professionals face challenges with a youth at-risk case. What would it be called, and what would this team do?
Preconditions to collaborate	- In your opinion, what is needed in collaboration between stakeholders ensure that youth and families with the most complex issues receive appropriate, cohesive support?
Taks and role division	- How are the tasks and responsibilities coordinated between the municipality and organizations in practice? - Is it clear who has which task in collaboration between stakeholders?
Responsibility	- How does the responsibility of other stakeholders relate to the responsibility of the municipality? - To what extent do you experience shared responsibility in collaboration with stakeholders in practice? - Do you find it important to experience this?
Trust expertise	- To what extent do you have trust in the expertise of stakeholders? - Why is that? - To what extent do you perceive trust in your own expertise from these stakeholders? - Why is that?
Professional autonomy	- To what extent does the municipality have the capacity to act on the advice of the multidisciplinary specialist team? For example, when it comes to funding custom solutions instead of purchased care - To what extent do contracted care providers have the capacity to implement custom solutions?
Reflection interview	- Finally, would you like to add anything? - How do you feel about the interview?

3.
